# The Effects of Seleno-Methionine in Cadmium-Challenged Human Primary Chondrocytes

**DOI:** 10.3390/ph17070936

**Published:** 2024-07-12

**Authors:** Valentina Urzì Brancati, Federica Aliquò, José Freni, Alice Pantano, Erika Galipò, Domenico Puzzolo, Letteria Minutoli, Herbert Ryan Marini, Giuseppe Maurizio Campo, Angela D’Ascola

**Affiliations:** 1 Department of Clinical and Experimental Medicine, University of Messina, Via Consolare Valeria 1, 98125 Messina, Italy; valeurzi@hotmail.it (V.U.B.); alicepantano097@gmail.com (A.P.); erikagalipo@gmail.com (E.G.); hrmarini@unime.it (H.R.M.); giuseppe.campo@unime.it (G.M.C.); angela.dascola@unime.it (A.D.); 2 Department of Biomedical and Dental Sciences and Morphofunctional Imaging, University of Messina, Via Consolare Valeria 1, 98125 Messina, Italy; faliquo@unime.it (F.A.); jofreni@unime.it (J.F.); puzzolo@unime.it (D.P.)

**Keywords:** apoptosis, cadmium, chondrocyte, potentially toxic element, seleno-methionine

## Abstract

Cadmium (Cd) is a potentially toxic element able to interfere with cellular functions and lead to disease or even death. Cd accumulation has been demonstrated in cartilage, where it can induce damage in joints. The aim of this study was to evaluate the effect of CdCl_2_ on primary cultures of human chondrocytes and the possible protective effect of seleno-methionine (Se-Met). Human primary articular chondrocytes were cultured and treated as follows: control groups, cells challenged with 7.5 μM and 10 μM CdCl_2_ alone, and cells pretreated with 10 and 20 μM Se-Met and then challenged with 7.5 μM and 10 μM CdCl_2_. Twenty-four hours after incubation, cell viability, histological evaluation with hematoxylin–eosin stain, and terminal deoxynucleotidyl transferase dUTP nick end labeling (TUNEL) assay were performed. Furthermore, reverse transcription-PCR was carried out to evaluate mRNA levels of *BAX*, *BAK1*, *CASP-3*, and *CASP-9*. After CdCl_2_ challenge at both doses, a reduced cell viability and an overexpression of *BAX*, *BAK1*, *CASP-3*, and *CASP-9* genes, as well as a high number of TUNEL-positive cells, were demonstrated, all parameters becoming higher as the dose of CdCl_2_ was increased. The pretreatment with Se-Met lowered the expression of all considered genes, improved cell viability and morphological changes, and reduced the number of TUNEL-positive cells. It was concluded that Se-Met plays a protective role against CdCl_2_-induced structural and functional changes in chondrocytes in vitro, as it improved cell viability and showed a positive role in the context of the apoptotic pathways. It is therefore suggested that a translational, multifaceted approach, with plant-based diets, bioactive functional foods, nutraceuticals, micronutrients, and drugs, is possibly advisable in situations of environmental pollution caused by potentially toxic elements.

## 1. Introduction

Cadmium (Cd) is a potentially toxic element (PTE) [1] present in the environment because of natural and anthropogenic processes. It is therefore able to occur in the contexts of water, air, and soil pollution, resulting in its entrance and accumulation in the food chain [2]. As a result, Cd can pass into the human body through inhalation from the environment or following cigarette smoking, or by eating or drinking contaminated food or water. Moreover, Cd can penetrate into the body following occupational exposure, owing to its use in industries associated with anticorrosive materials, plastic stabilizers, electronic devices, batteries, and paint pigments [3]. Once absorbed, it can reach different organs through blood circulation, where about 90% of Cd is bound to α2-macroglobulin and albumin in serum [4], compromising human health [5].

Cd is potentially toxic even under low-dose exposure conditions [6,7]. It is not required in any mammalian physiological process, but it can mimic essential metal ions by competing with their transporters, or replacing them in intracellular macromolecules, thus interfering with cellular functions and leading to disease or even death [8]. As its half-life is 16–30 years [9], the International Agency for Research on Cancer and the USA National Toxicology Program have indicated that Cd is a type I carcinogen, being related to higher risks of lung, breast, liver, colon, and genitourinary cancers [10].

Several in vitro studies have assessed Cd toxicity in different cellular lines, such as normal mouse renal MM55.K cells [11], duck renal tubular epithelial cells [12], human renal proximal-tubule epithelial cells (RPTEC/TERT1) [13], human liver carcinoma HepG2 and human normal liver THLE-3 cells [14], alpha mouse liver 12 (AML12) cells [15], SN56 cholinergic neurons [16], PC12 cells and primary rat cerebral cortical neurons [17,18], and cerebellar cells [19]. Cd toxicity has been linked as a cause to oxidative stress, endoplasmic reticulum stress, and apoptosis. In more detail, these studies have reported an inhibitory effect of Cd on antioxidant enzymes, one of which causes reactive oxygen species (ROS) production [14,19]; endoplasmic reticulum stress [11]; oxidative stress-mediated apoptosis with increased Bax, Bak-1, and Caspase-3 expression [12]; activation of the NLRP3 inflammasome [13]; and inhibition of the autophagic flux [15]. Cd also has caused cholinergic neuron death by blocking muscarinic acetylcholine receptor (M1) [16], initiated Fas/FasL-mediated mitochondrial apoptotic pathways in neuronal cells [17], and caused mitochondrial dysfunction via ROS-mediated Sirtuin 1 (SIRT1) suppression [18].

In the body, Cd is deposited mainly in the kidney, testis, brain, liver, and bones [20,21]. Furthermore, Cd accumulation has also been demonstrated in cartilage, enabling it to induce damage in joints [22]. In fact, in vivo, Cd is able to induce in mice a significant reduction, in comparison to control animals, in cartilage volume and surface area, as well as number of chondrocytes [23], and a positive correlation between its concentration in the body and arthritis has been shown [24].

In this context, the possibility of relieving the negative effects of Cd on chondrocytes could be of particular interest. A protective role of selenium (Se) against Cd toxicity has been demonstrated in vivo [25,26,27,28]. Among other trace elements, Se can be found both in inorganic species, such as SeO_3_^2−^ and SeO_4_^2−^, and in organic forms, such as amino acids like selenocysteine and seleno-methionine (Se-Met), or proteins like selenoalbumin and antioxidase, thus playing a significant biological role in living organisms [29,30,31,32]. In fact, Se is involved in the balance of redox reactions, in strengthening the immune system, and in detoxification processes [33].

However, whether Se reduces the toxic effect of Cd on chondrocytes in vitro remains uncertain; therefore, the role of a pretreatment of Se-Met before cadmium chloride (CdCl_2_) addition in primary cultures of human chondrocytes, with particular regard to cell viability and apoptosis, was evaluated. This line of research hopes to add a novel aspect to the translational multifaceted approaches (i.e., healthy dietary habits, nutraceuticals, medical foods, micronutrients, pharmaceuticals, and drugs) employed against environmental pollution caused by PTEs such as Cd.

## 2. Results

### 2.1. Effects of Se-Met on CdCl_2_-Induced Cell Injury

**Figure 1:**
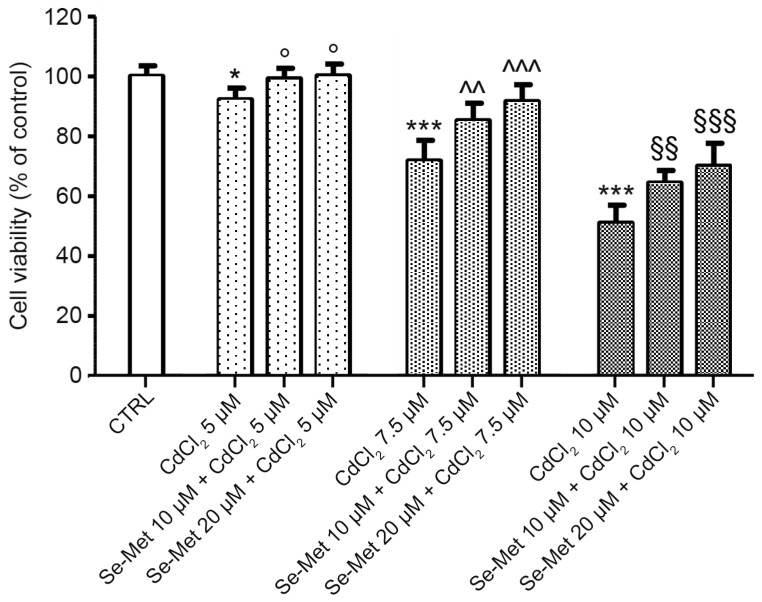
Evaluation of cell viability in control chondrocytes and in chondrocytes pre-treated with Se-Met before CdCl_2_ addition. Control chondrocytes, from culture medium alone, or from culture medium supplemented with only 10 μM Se-Met, and 20 μM Se-Met. Control groups showed normal viability, and control data were incorporated and presented as a single value. Values are the mean ± SD of no less than five experiments and are expressed as the % increase versus controls (CTRL). * *p* < 0.05 and *** *p* < 0.001 vs. CTRL; ° *p* < 0.05 vs. 5 μM CdCl_2_; ^^ *p* < 0.01 and ^^^ *p* < 0.001 vs. 7.5 μM CdCl_2_; ^§§^ *p* < 0.01 and ^§§§^ *p* < 0.001 vs. 10 μM CdCl_2_.

CdCl_2_ significantly reduced chondrocyte viability in a dose-dependent manner, mostly at concentrations of 7.5 and 10 μM (Figure 1; *** *p* < 0.001 vs. CTRL). The pretreatment with Se-Met before CdCl_2_ addition partially restores cell viability compared to cells incubated with CdCl_2_ alone, at each of the studied concentrations (5, 10, and 20 μM), in a dose-dependent manner (Figure 1; ° *p* < 0.05 vs. 5 μM CdCl_2_; ^^ *p* < 0.01 and ^^^ *p* < 0.001 vs. 7.5 μM CdCl_2_; ^§§^ *p* < 0.01 and ^§§§^ *p* < 0.001 vs. 10 μM CdCl_2_).

### 2.2. Se-Met Preserves Chondrocytes from the Damage Induced by CdCl_2_

**Figure 2:**
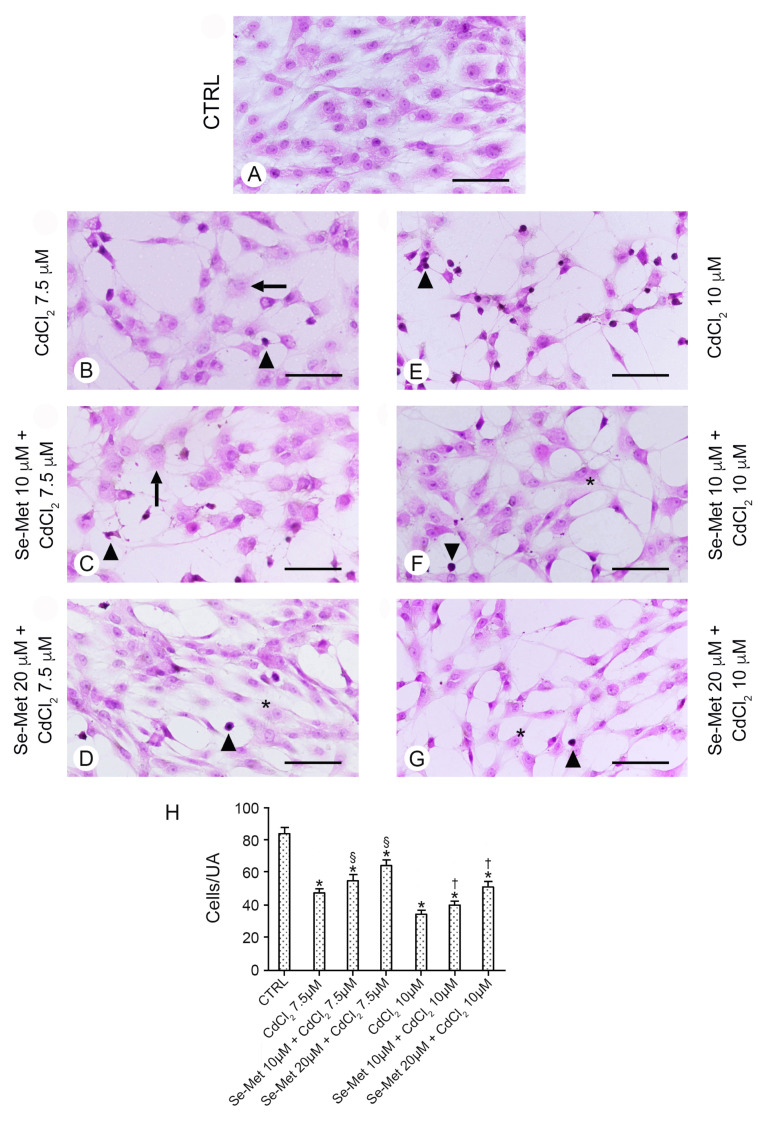
Histological organization of the different groups of chondrocytes evaluated with hematoxylin–eosin stain. (Scale bar: 100 μm). (**A**) Control chondrocytes, from culture medium alone, or from culture medium supplemented with only 10 μM Se-Met, and 20 μM Se-Met. All control groups showed normal morphology, and control data were incorporated and presented as a single image. In all controls, cells show uniform size, regular distribution, many processes, and euchromatic nuclei. (**B**) Chondrocytes treated with 7.5 μM CdCl_2_ alone. A lower number of cells is present; normal, necrotic cells (arrow), and cells with heterochromatic nuclei (arrowhead) are evident. (**C**) Chondrocytes pre-treated with 10 μM Se-Met plus 7.5 μM CdCl_2_. A mild increase in cells/UA is observed, but normal, necrotic cells (arrow), and cells with heterochromatic nuclei (arrowhead) are still present. (**D**) Chondrocytes pre-treated with 20 μM Se-Met plus 7.5 μM CdCl_2_. A significant increase in cells/UA is observed; many of them are elongated (*) and show thin processes, even if some have heterochromatic nuclei (arrowhead). (**E**) Chondrocytes treated with 10 μM CdCl_2_ alone. Only a few normal cells are present; the other are small, with heterochromatic nuclei and thin, elongated processes (arrowhead). (**F**) Chondrocytes pre-treated with 10 μM Se-Met plus 10 μM CdCl_2_. The largest number of the cells show a close-to-normal morphology (*), but some cells with heterochromatic nuclei (arrowhead) are evident. (**G**) Chondrocytes pre-treated with 20 μM Se-Met plus 10 μM CdCl_2_. Normal cells with thin processes (*) and occasional cells with heterochromatic nuclei (arrowhead) are observed. (**H**) Histogram of the cells/UA in the different chondrocyte groups (mean ± SD). * *p* < 0.05 versus CTRL; § *p* < 0.05 versus CdCl_2_ 7.5 μM alone; † *p* < 0.05 versus CdCl_2_ 10 μM alone.

The control chondrocytes, which received no CdCl_2_, were those from (i) culture medium alone, or from culture medium supplemented with (ii) 10 μM Se-Met, and (iii) 20 μM Se-Met alone. All control groups showed cells with normal morphology. For this reason, for sake of simplification, data from (i), (ii), and (iii) were combined, and they are presented in all figures as a single image. In all controls, cells showed uniform size, regular distribution, many processes, and euchromatic nuclei (Figure 2A). In chondrocytes treated with 7.5 μM CdCl_2_ (Figure 2B), a statistically significant reduction in their number was observed (Figure 2H; * *p* < 0.05 versus CTRL) and normal or necrotic cells together with cells with heterochromatic nuclei were present. When these chondrocytes were pre-treated with 10 μM Se-Met, a mild increase in cells/UA was observed (Figure 2H; * *p* < 0.05 versus CTRL; § *p* < 0.05 versus CdCl_2_ 7.5 μM alone), bur their morphology was not improved (Figure 2C). If instead chondrocytes pre-treated with 20 μM Se-Met and then challenged with 7.5 μM CdCl_2_ were considered, a significant increase in cells/UA was observed (Figure 2H; * *p* < 0.05 versus CTRL; § *p* < 0.05 versus CdCl_2_ 7.5 μM alone) and the presence of elongated cells with thin processes was observed; however, some cells with heterochromatic nuclei were still present (Figure 2D). In chondrocytes treated with 10 μM CdCl_2_ alone, a sharp reduction in cells/UA was demonstrated (Figure 2H; * *p* < 0.05 versus CTRL). Only a few normal cells were present, while the others were small, with heterochromatic nuclei and thin, elongated processes (Figure 2E). The pretreatment with 10 μM Se-Met induced a significant increase in cells/UA (Figure 2H; * *p* < 0.05 versus CTRL, † *p* < 0.05 versus CdCl_2_ 10 μM alone) and the maintenance of a close-to-normal morphology of a large part of the cells (Figure 2F). If instead chondrocytes were pre-treated with 20 μM Se-Met, the absolute number of cells/UA was increased (Figure 2H; * *p* < 0.05 versus CTRL, † *p* < 0.05 versus CdCl_2_ 10 μM alone) and both elongated cells with thin processes and small cells with heterochromatic nuclei were observed (Figure 2G).

### 2.3. Se-Met Effects on Transcription of Apoptotic Genes

**Figure 3:**
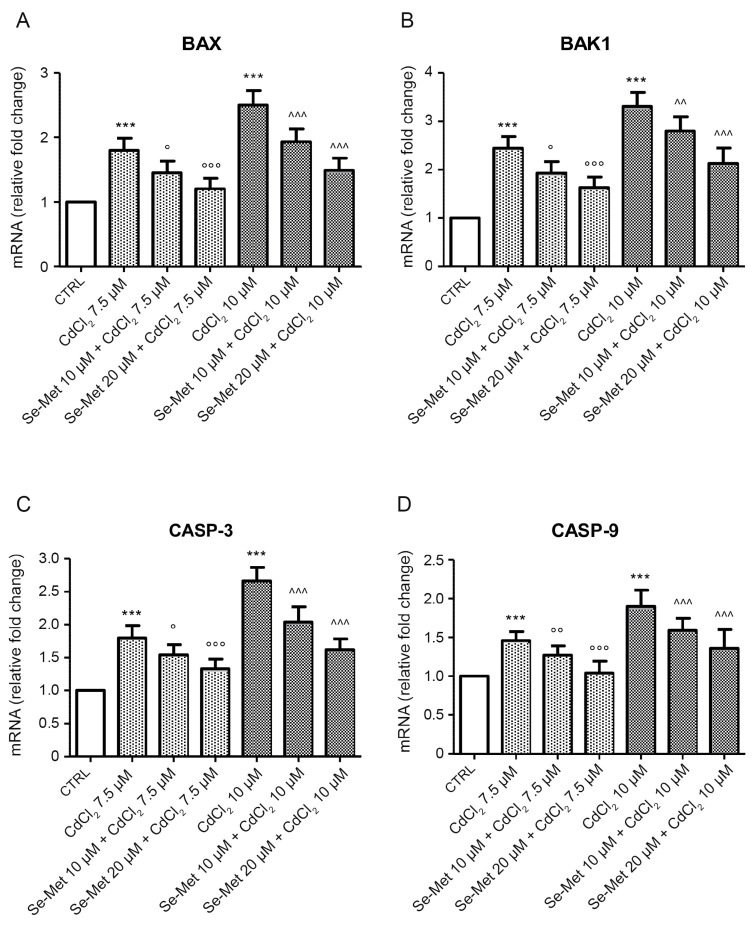
Evaluation of mRNA levels of *BAX* (**A**), *BAK1* (**B**), *CASP-3* (**C**), and *CASP-9* (**D**) in control chondrocytes and in chondrocytes pre-treated with Se-Met before CdCl_2_ addition. Control chondrocytes, from culture medium alone, or from culture medium supplemented with only 10 μM Se-Met, and 20 μM Se-Met showed similar mRNA levels of apoptotic genes and data were incorporated and presented as a single value. Values are the mean ± S.D. of no less than five experiments and are expressed as fold change with respect to controls. *** *p* < 0.001 vs. CTRL; ° *p* < 0.05, °° *p* < 0.01 and °°° *p* < 0.001 vs. 7.5 μM CdCl_2_ alone; ^^ *p* < 0.01 and ^^^ *p* < 0.001 vs. 10 μM CdCl_2_ alone.

The expression of pro-apoptotic genes *BAX*, *BAK1*, *CASP-3* and *CASP-9* in cultured cells was evaluated to assess the effect of Se-Met on CdCl_2_-induced apoptosis. CdCl_2_ was able to increase mRNA levels of all genes, in a dose-dependent manner (Figure 3; *** *p* < 0.001 vs. CTRL). The addition of Se-Met decreased the upregulation promoted by CdCl_2_, showing a protective effect at both doses (Figure 3; ° *p* < 0.05, °° *p* < 0.01 and °°° *p* < 0.001 vs. 7.5 μM CdCl_2_ alone; ^^ *p* < 0.01 and ^^^ *p* < 0.001 vs. 10 μM CdCl_2_ alone).

### 2.4. Se-Met Effects on Apoptosis with TUNEL Assay

**Figure 4:**
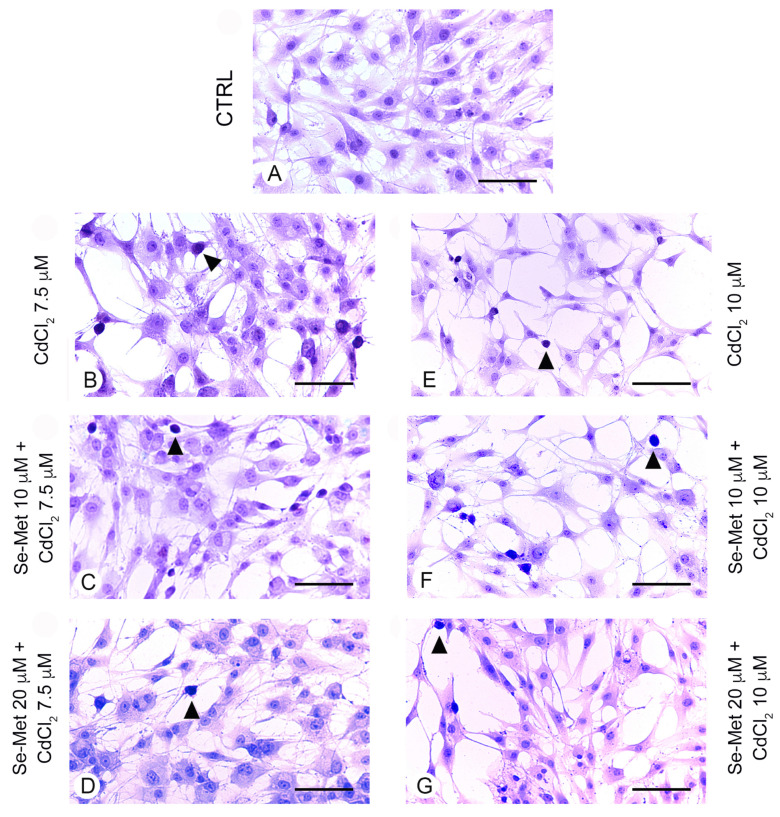

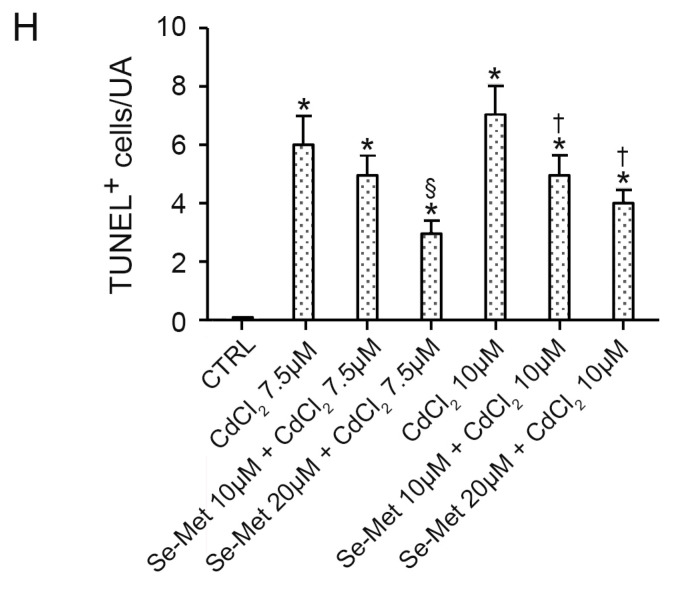
Assessment of apoptosis in the different groups of cells with TUNEL technique (scale bar: 100 μm). (**A**) Control chondrocytes, from culture medium alone, or from culture medium supplemented with only 10 μM Se-Met, and 20 μM Se-Met. All groups showed similar morphology, and control data were combined and presented as a single image. No TUNEL-positive chondrocytes are observed. (**B**) In chondrocytes challenged with 7.5 μM CdCl_2_ alone, the number of positive cells (arrowhead) is significantly high versus controls. (**C**) After a pretreatment with 10 μM Se-Met, TUNEL-positive chondrocytes (arrowhead) challenged with 7.5 μM CdCl_2_ show a mild, but statistically unsignificant reduction versus CdCl_2_ alone. (**D**) Chondrocytes pretreated with 20 μM Se-Met and challenged with 7.5 μM CdCl_2_. An evident and statistically significant reduction in TUNEL-positive cells (arrowhead) is present. (**E**) Chondrocytes challenged with 10 μM CdCl_2_ alone. TUNEL-positive cells (arrowhead) are significantly more numerous versus controls. (**F**) In chondrocytes pretreated with 10 μM Se-Met and challenged with 10 μM CdCl_2_, a significant reduction in TUNEL-positive cells (arrowhead) is observed. (**G**) In chondrocytes pretreated with 20 μM Se-Met and challenged with 10 μM CdCl_2_, a significant reduction in TUNEL-positive cells (arrowhead) is demonstrated. (**H**) Histogram of TUNEL-positive cells/UA in the different groups of chondrocytes (mean ± SD). * *p* < 0.05 versus CTRL; § *p* < 0.05 versus 7.5 μM CdCl_2_ alone; † *p* < 0.05 versus 10 μM CdCl_2_ alone.

No positive cells were observed in chondrocytes from all control groups with TUNEL assay (Figure 4A). In chondrocytes challenged with CdCl_2_ alone at the concentration of 7.5 μM, a significantly high number of positive cells was observed (Figure 4B,H; * *p* < 0.05 versus CTRL). When chondrocytes challenged with CdCl_2_ at the concentration of 7.5 μM were pretreated with Se-Met at a dose of 10 μM, a mild, but statistically unsignificant reduction in TUNEL-positive chondrocytes versus CdCl_2_ alone was demonstrated (Figure 4C,H; * *p* < 0.05 versus CTRL). The pretreatment with Se-Met at a dose of 20 μM to chondrocytes challenged with CdCl_2_ at the same concentration of 7.5 μM induced an evident and significant reduction in TUNEL-positive cells (Figure 4D,H; * *p* < 0.05 versus CTRL; § *p* < 0.05 versus 7.5 μM CdCl_2_ alone). In chondrocytes challenged with CdCl_2_ alone at the concentration of 10 μM, the number of TUNEL-positive cells was high and statistically significant versus controls (Figure 4E,H; * *p* < 0.05 versus CTRL). When chondrocytes challenged with CdCl_2_ at the concentration of 10 μM were pretreated with Se-Met at a dose of 10 μM, an evident and significant reduction in the number of TUNEL-positive cells was observed (Figure 4F,H; * *p* < 0.05 versus CTRL; † *p* < 0.05 versus 10 μM CdCl_2_ alone). The pretreatment with Se-Met at a dose of 20 μM of chondrocytes challenged with 10 μM CdCl_2_ was able to induce an evident and statistically significant reduction in TUNEL-positive cells, even if lower when compared to chondrocytes of Se-Met 20 μM plus CdCl_2_ 7.5 μM group (Figure 4G,H; * *p* < 0.05 versus CTRL; † *p* < 0.05 versus 10 μM CdCl_2_ alone).

## 3. Discussion

Cd is a PTE [1], able to induce damage in most tissues and organs, including cartilage [24], where it induces oxidative stress with changes in composition and function [34]. On this basis, the possible role of the pretreatment with Se-Met, a well-known micronutrient with multiple positive effects on cell health [35], has been tested in the present in vitro model to suggest novel translational multifaceted approaches against environmental pollution caused by PTEs, such as Cd.

Cd toxicity derives mainly from its inhibitory effects on antioxidant enzymes, such as glutathione reductase [36] and superoxide dismutase [37]. In fact, it binds with high-affinity -SH and disulfide -S-S- groups, negatively interfering with the functions of the biological structures containing these chemical groups. As a result, Cd causes oxidative stress and ROS production, inducing oxidation and degradation of proteins, DNA, lipids, and phospholipids of the cellular membrane and triggering cell apoptosis and necrosis. Furthermore, Cd affects oxidative phosphorylation and ATP synthesis, inhibiting the mitochondrial electron transport chain [8].

Few studies are, however, available on the effect of Cd on chondrocytes in in vitro studies. Fernández-Torres et al. [38] showed that Cd can negatively influence the viability of chondral cells. Zamudio-Cuevas et al. [39], in micromass cultures of human chondrocytes exposed to Cd, showed loss of collagen II, aggrecan, proteoglycans, and glycosaminoglycans in the extracellular matrix, increased expression of metalloproteinases (MMP), and activation of Interleukin (IL)-1β and IL-6. Recently, in chondrocytes obtained from chicken embryos, Gu et al. [40] demonstrated toxic effects of Cd, such as an increased expression of proapoptotic Bax and of MMP-9, and a reduced expression of collagen IIα1 and acid mucopolysaccharides.

It was also observed that functional changes of cartilage induced by Cd can be a risk factor, in addition to genetics, obesity, sex, and age, for the evolution of joint diseases, such as osteoarthritis (OA), as it interferes with the uptake of essential elements necessary for cartilage balance between matrix synthesis and degradation [41,42].

In the present experimental model, a dose–response analysis of different concentrations of CdCl_2_ (2.5, 5, 7.5, 10, 12.5, 15 μM), some based on a previous paper [39], was performed, demonstrating that the intermediate doses of 7.5 and 10 μM induced changes in chondrocytes.

Among the mechanisms involved in Cd toxicity, apoptosis plays an important role. Chondrocytes are present only in cartilage, and, owing to their low or absent reproductive capacity, their response to Cd-induced apoptosis is critical, as it causes irreversible damages in the extracellular matrix [43]. As an apoptotic effect on chondrocytes induced by Cd has not been yet demonstrated, this topic was evaluated in human chondrocyte cultures. The results showed a peculiar involvement of the pathways of apoptosis after CdCl_2_ challenge. In particular, an overexpression of *BAX*, *BAK1*, *CASP-3*, *CASP-9* genes and an elevated number of TUNEL-positive cells were demonstrated, which was higher as the dose of CdCl_2_ increased. In agreement with similar findings reported by other authors [44,45,46], these results confirmed the induction of apoptosis by Cd, indicating the activation of the intrinsic pathway.

In order to counteract the apoptosis induced by Cd, many pharmacological approaches were performed in many tissues [47,48,49,50,51,52,53]. In particular, the protection of chondrocytes against the toxic action of Cd can be oriented towards the use of natural substances able to prevent the apoptosis of cultured cells.

Among them, Se, an essential trace element, showed protective action against Cd toxicity both in vivo [54] and in vitro [55] in all its forms (selenite, nanoSe, Se-Met) [56]. In particular, Se showed a significant antagonistic effect on apoptosis in chicken ovary [57] and positively regulated anti-apoptosis pathways in TM3 cells [58]. Se forms complexes with Cd, thus preventing its accumulation in tissues [56], increases antioxidant selenoprotein levels, such as glutathione peroxidase, catalyzing the reaction between glutathione and hydrogen peroxide, and reduces malondialdehyde levels [56].

In cartilage, Se influences the metabolism of extracellular matrix [59] and, when deficient, it can be involved in an osteochondropathy, Kashin–Beck disease [60].

Since no data are available as to the current knowledge on the possible protective role of Se, and of Se-Met in particular, against Cd-induced toxicity in cartilage, the effects of the interaction between Se-Met and CdCl_2_ in human chondrocytes were explored.

Exposure of chondrocyte cultures to different concentrations of CdCl_2_ had a negative impact on cell viability, as demonstrated by the significant decrease in the number of viable cells showed by MTT and histological evaluation. Se-Met had a protective role, as it restored cell viability in chondrocytes pretreated with different concentrations of Se-Met before CdCl_2_, compared to those treated with CdCl_2_ alone, thus demonstrating a positive protective role in cell viability and an improvement of the structural organization of chondrocytes after the negative impact of PTEs, such as Cd.

Biochemical, molecular, and morphometric analyses were performed to confirm the positive role of Se-Met pretreatment in cell viability and structural organization in the adverse impact of Cd challenge. The CdCl_2_-induced increased apoptosis was significantly counteracted by the pretreatment with Se-Met, as demonstrated by the reduction in the intrinsic pathway of apoptosis, especially *BAX* and *BAK1*, involved in the permeabilization of mitochondrial membrane [61], and of the effector proteases, particularly *CASP-3* and *-9*, responsible for initiating the degradation phase of apoptosis [62]. These antiapoptotic effects could also be partially justified by an overlapping between apoptosis and inflammation; in fact, a possible involvement of methionine in lowering induced inflammatory response and oxidative status has been previously demonstrated [63]. In turn, a lower percentage of TUNEL-positive cells was observed, indicating that Se-Met also has a significant positive role in modulating apoptosis in chondrocytes.

Overall, on the basis of the results of these experiments, the following strengths were highlighted. First, it is confirmed that trace elements, such as Se, have a crucial role in health and diseases [64]; indeed, in the present experimental model, the organic form Se-Met effectively modulated the apoptosis in human chondrocytes challenged with an extremely toxic industrial and environmental pollutant, such as Cd. Second, to our knowledge, this paper revealed for the first time a protective effect of Se-Met pretreatment against CdCl_2_ in human chondrocytes and better defined the dose–response effect; as for this purpose, it is important to underline, for a broad audience of readers and more in general for researchers interested to this specific topic, that, generally, the cartilage was not deeply investigated and, even more, after Cd exposure. As a matter of fact, our research group has demonstrated the effects of several compounds as a possible challenge against PTEs, such as Cd, in different organs [4,20,65], even if they do not represent common targets, showing the possible protective role against Cd toxicity, with a careful view to translational science.

Of course, in this context, it should be carefully focused, “keeping feet on the ground” and without generating easy enthusiasm, on a synergistic or antagonistic action between different micronutrients, such as Se, bioactive foods, and/or nutraceuticals of plant-based diets, on neuroendocrine immune system modulation and gut microbiota dysbiosis, with particular regard to the presence of environmental pollution caused by PTEs, such as Cd [66,67].

In the present experimental model, an additional limit is clearly represented by the fact that the results obtained in vitro are inevitably preliminary, even if undoubtedly encouraging, and open further investigations experimentally in vivo and in patients. Moreover, it is currently impossible to demonstrate whether the organic form Se-Met effectively modulates the apoptosis in response to CdCl_2_ challenge in cotreatment or in post-treatment, and the possible recommended doses to protect the human body.

Overall, a large body of literature, also including personal previous experimental observations [27,68], suggests that the effect of healthy dietary strategies, as well as the multifaceted mechanism of action of Se alone or in combination with other nutraceuticals/functional foods [26,27,64,69], could effectively counteract the detrimental molecular cascade in organ injury, also including the cartilage, caused by environmental PTEs, such as Cd.

It is hoped that the present experiments, despite the above limitations and the lack of clinical data available, could be helpful in improving the quality of life, as well as environmental and food sustainability, particularly in under-developed countries. The natural presence and/or the addition to the diet of compounds such as Se can be considered a new trustworthy medical tactic in subjects exposed to PTEs, in particular to those whose mechanisms of action are similar to Cd [70], keeping in mind that pathologies are multifactorial events which therefore require approaches aimed at different targets simultaneously. Moreover, another translational implication of the present data is to test whether the organic form of Se, Se-Met (alone or in combination with other compounds), may have prophylactic and/or therapeutic effects against the chondrocyte alterations caused by exposure to a number of cartilage-disrupting chemicals agents, such as organochlorine compounds, pesticides, bisphenol A, nitrates [71,72,73].

## 4. Materials and Methods

### 4.1. Cell Culture

Human primary articular chondrocytes were obtained from ScienceCell^TM^ Research Laboratories (Carlsbad, CA, USA). Cells were cultured in 75 cm^2^ flasks containing 15 mL of a specific Chondrocyte Medium, with the addition of 2.5% fetal bovine serum (FBS), L-glutamine (2.0 mM), and penicillin/streptomycin (100 U mL^−1^, 100 mg mL^−1^). All products were provided by ScienceCell^TM^ Research Laboratories (Carlsbad, CA, USA). Chondrocytes were incubated at 37 °C in humidified air with 5% CO_2_ [74]. Experiments were performed using chondrocyte cultures between the third and the fifth passage.

### 4.2. Cell Treatment

Chondrocytes were cultured in 24-well culture plates at a density of 5 × 10^4^ cells/well. Twelve hours after plating (time 0), the culture medium was replaced with fresh medium containing 1% FBS and Se-Met (Sigma-Aldrich, Milan, Italy) was added at doses of 5, 10, and 20 μM [68]. After incubating overnight, CdCl_2_ was added at doses of 5, 7.5, and 10 μM [39]. Finally, 24 h later, chondrocytes were subjected to gene expression evaluation and MTT assay. To perform histological evaluation and terminal deoxynucleotidyl transferase dUTP nick end labeling (TUNEL) assay, further experiments were conducted using LabTech^TM^ 8-Chamber Slide Systems (Thermo Fisher Scientific, Waltham, MA, USA), where chondrocytes were seeded at a density of 1 × 10^4^ cells/well. The Se-Met and CdCl_2_ concentrations were established by preliminary tests assessing their effects on cell vitality at increasing concentrations (see Supplementary Materials).

### 4.3. MTT Assay

Cell viability was evaluated by the colorimetric assay that uses the 3-[4,5-dimethylthiazol-2-yl]-2,5 diphenyl tetrazolium bromide (MTT) to measure cell metabolic activity [75]. The MTT is a yellowish solution and is converted to water-insoluble MTT-formazan of dark blue color by mitochondrial dehydrogenases of living cells. Twenty-four hours after incubation with CdCl_2_ in the presence/absence of Se-Met, MTT solution (5 mg/mL) was added to cultured cells and incubated for two hours at 37 °C. Subsequently the formazan crystals were dissolved with DMSO and absorbance was measured at 550 nm using a spectrophotometer (Biospectrometer basic, Eppendorf, Enfield, CT, USA). The viability was expressed as absorbance relative to control cells (% of untreated controls, considered 100%).

### 4.4. Histological Evaluation

Slides were fixed in 4% paraformaldehyde and then stained with hematoxylin and eosin (HE). They were then photographed with a Nikon Ci-L (Nikon Instruments, Tokyo, Japan) light microscope at a magnification of 200× with a digital camera Nikon DS-Ri2 [26].

### 4.5. Evidence of Apoptosis with TUNEL Assay

The TUNEL technique was performed with the apoptosis detection kit (In situ Apoptosis Detection kit, Abcam, Cambridge, UK) according to the manufacturer’s instructions. Briefly, the slides, once fixed in 4% paraformaldehyde and washed in PBS, were permeabilized with proteinase K. After blocking endogenous peroxidase activity with 3% H_2_O_2_ in methanol, the slides were incubated with terminal deoxynucleotidyl transferase, with biotin-labeled deoxynucleotides, with streptavidin-horseradish peroxidase conjugate, and lastly with diaminobenzidine solution. For a better evaluation of apoptotic cells, a rapid counterstaining with hematoxylin was performed. Slides were then photographed with the digital camera Nikon Ds-Ri2 of the Nikon Ci-L light microscope [27].

### 4.6. Morphometric Evaluation

Once saved as Tagged Image Format Files (TIFF), the images were blindly evaluated by two trained observers. As to the slides stained with HE, a Unit Area (UA = 250 × 250 μm) was chosen to calculate the mean number of cells/UA from 20 UAs for each group, including in the evaluation the cells in contact with the right and the top borders. On the contrary, the cells contacting the left and the bottom borders were not counted. For the evaluation of apoptosis, the mean number of TUNEL-positive cells/UA (UA = 250 × 250 μm) was obtained from 20 UAs for each group. The values of TUNEL-positive cells/UA were obtained as indicated above [27].

### 4.7. RNA Extraction and Quantitative Reverse Transcription-PCR (qRT-PCR)

Total RNA was isolated from the chondrocytes using Total Purification Plus Kit (Norgen Biotek Corporation, Thorold, ON, Canada). After quantification, total RNA was reverse transcribed using the high-capacity reverse transcription kit (Applied Biosystems, Foster City, CA, USA), following the manufacturer’s protocols. qRT-PCR reactions were performed by using PowerUp SYBR Master Mix (Thermo Fisher Scientific, Waltham, MA, USA) in order to evaluate gene expression of *CASP-3*, *CASP-9*, *BAX*, *BAK1*, and *β-actin*. Reactions were performed by using a 7500 Real-Time PCR System model 7500 (Applied Biosystems, Foster City, CA, USA). β-actin mRNA was used as an endogenous control to allow relative quantification of mRNAs. After normalization, the mean value of untreated chondrocyte target gene levels was chosen as the calibrator and the results were expressed according to the 2^−ΔΔCt^ calculation, as fold change relative to normal controls [75]. Primers used for target and endogenous control genes are listed in Table 1. Specific primers were designed based on the published nucleotide sequence of the NCBI GenBank database.

### 4.8. Statistical Analysis

All groups were evaluated with the one-way ANOVA followed by the Newman–Keuls post-test for intergroup comparisons. A *p*-value of ≤0.05 was considered statistically significant. Values are indicated as mean ± standard deviation (SD).

## 5. Conclusions

In conclusion, on the basis of these results, it is suggested that Se-Met might offer a new possible approach that, appropriately combined with good agricultural practice to abate Cd contamination in food crops and animals, could also provide an intriguing preventive and/or therapeutic strategy to counteract Cd-induced cartilage injury. Therefore, recent research on multifaceted approaches with plant-based diets, bioactive functional foods, nutraceuticals, micronutrients, and drugs can be considered a new therapeutic tool to prevent and treat NCDs and their comorbidities, even with randomized controlled clinical trials on widely exposed populations.

## Figures and Tables

**Table 1:** Sequences of primers used for qRT PCR.

Gene	Sequence
*β-actin*	Fw: 5′-AGAGCTACGAGCTGCCTGAC-3′
	Rw: 5′-AGCACTGTGTTGGCGTACAG-3′
*BAX*	Fw: 5′-GACGAACTGGACAGTAACATGG-3′
	Rw: 5′-TCAGAAAACATGTCAGCTGCC-3′
*BAK1*	Fw: 5′-TGGGACACTGTGAACCAGGA-3′
	Rw: 5′-GAGGAAGCCAAACACCAGTAGG-3′
*CASP-3*	Fw: 5′-TGAGGCATGGTGAAGAAGGA-3′
	Rw: 5′-TCCAGTTCTGTACCACGGCA-3′
*CASP-9*	Fw: 5′-CTGGACGCCATATCTAGTTTGC-3′
	Rw: 5′-AACGTACCAGGAGCCACTCTT-3′

## Data Availability

The data presented in this study are available on request to the corresponding author.

## Supplementary Materials

The following supporting information can be downloaded at: https://www.mdpi.com/article/10.3390/ph17070936/s1, Figure S1: Dose-response effects of Se-Met on chondrocytes viability. Values show the mean ± S.D. of no less than five experiments and are expressed as *%* increase compared to controls. * *p* < 0.05 vs CTRL; Figure S2: Dose-response effects of CdCl_2_ on chondrocytes viability. Values show the mean ± S.D. of no less than five experiments and are expressed as % increase compared to controls. * *p* < 0.05 and *** *p* < 0.001 vs CTRL; Figure S3: Histological organization of chondrocytes exposed to different doses of CdCl_2_ and evaluated with hematoxylin-eosin stain (Scale bar: 100 μm). **A**: Control groups. Chondrocytes are regularly distributed and show uniform size, euchromatic nuclei and long processes. **B**: Chondrocytes challenged with 2.5 μM CdCl_2_. Cells show irregular size and, occasionally, foamy cytoplasm (arrow). **C**: Chondrocytes challenged with 5 μM CdCl_2_. Normal or necrotic (arrow) cells or cells with reduced size and heterochromatic nuclei (arrowhead) are present. **D**: Chondrocytes challenged with 7.5 μM CdCl_2_. Cells are reduced in number, and normal, necrotic (arrow) or small with heterochromatic nuclei (arrowhead) cells are evident. **E**: Chondrocytes challenged with 10 μM CdCl_2_. A lower number of cells is present. Few are normal, the other are small, with heterochromatic nuclei and thin processes (arrowhead). **F**: Histogram of chondrocytes/UA in the different groups (mean ± SD). * *p* < 0.05 versus CTRL.
