# Supplementary material for: The Effects of Seleno-Methionine in Cadmium-Challenged Human Primary Chondrocytes
Source: Pharmaceuticals (Basel). 2024 Jul 12;17(7):936. doi: 10.3390/ph17070936 (PMC11280455; doi:10.3390/ph17070936)
Supplement: Supplementary file 1 [file pharmaceuticals-17-00936-s001.zip › pharmaceuticals-3079616-supplementary.pdf]

# Supplementary Materials

*Effect of Se-Met and $CdCl_2$ on Cell Viability*

A dose-response experiment was performed to evaluate the effects of Se-Met on chondrocytes viability and to choose the concentrations for our experiments, adding Se-Met at doses: 2.5, 5, 10, 20, and 40 µM., $CdCl_2$ was added in other plates of chondrocytes cultures at doses: 2.5 5, 7.5, 10, and 12.5 µM. Cell viability was evaluated as in *Material and Methods.*

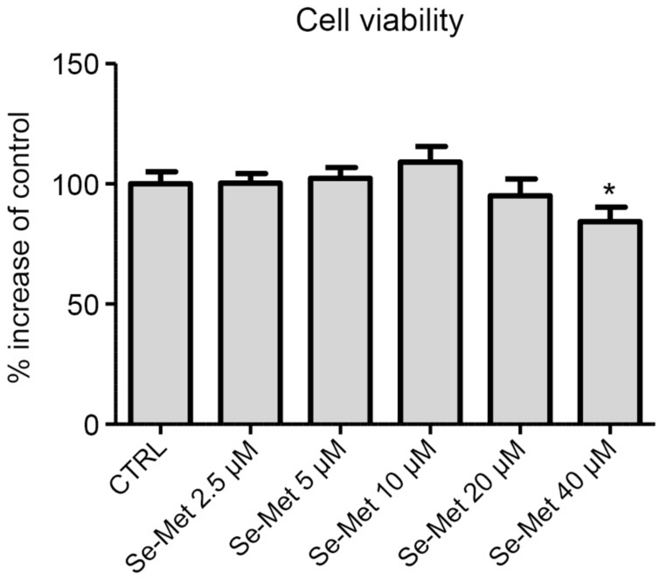


**Figure S1** - Dose-response effects of Se-Met on chondrocytes viability. Values show the mean ± S.D. of no less than five experiments and are expressed as % increase compared to controls. * $p < 0.05$ vs CTRL.

As reported in Figure S1, Se-Met does not interfere with viability except when used at the highest concentration, corresponding to 40 µM. Based on these findings, this dose was considered weakly toxic and excluded in all subsequent experiments.

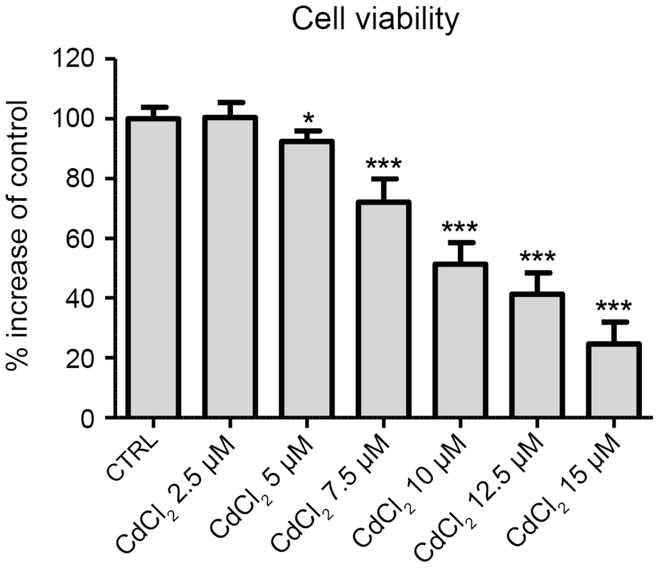


**Figure S2** - Dose-response effects of $CdCl_2$ on chondrocytes viability. Values show the mean ± S.D. of no less than five experiments and are expressed as % increase compared to controls. * $p < 0.05$ and *** $p < 0.001$ vs CTRL.

As reported in Figure S2, $CdCl_2$ reduced chondrocyte viability from the concentration of 5 µM, in a dose-dependent manner. As the concentration of 10 µM already reduced the viability of about 50%, we used concentrations ranging from 5 to 10 µM in all subsequent experiments.

*Effects of Different Doses of $CdCl_2$ on Chondrocytes*

Control cells (Figure S3A) were numerous, regularly distributed, and showed evident processes, euchromatic nuclei with nucleoli and uniform size. In chondrocytes from culture medium challenged with 2.5 µM $CdCl_2$ (Figure S3B), a mild, statistically unsignificant reduction of the cell number was observed (Figure S3F); however, cell size was irregular and some chondrocytes showed a foamy cytoplasm. In chondrocytes challenged with 5 µM $CdCl_2$ (Figure S3C), cells showed either euchromatic nuclei with evident nucleoli or heterochromatic, condensed nuclei; in addition, occasional necrotic cells were also present. In chondrocytes challenged with 7.5 µM $CdCl_2$ (Figure S3D), a statistically significant reduction of the cell number was observed (Figure S3F); the coexistence of few normal cells, necrotic cells and cells with heterochromatic nuclei was demonstrated. In chondrocytes challenged with 10 µM $CdCl_2$ (Figure S3E), a sharp reduction of cells/UA was present (Figure S3F); only few cells appeared normal, while they were generally small, with heterochromatic nuclei and thin processes.

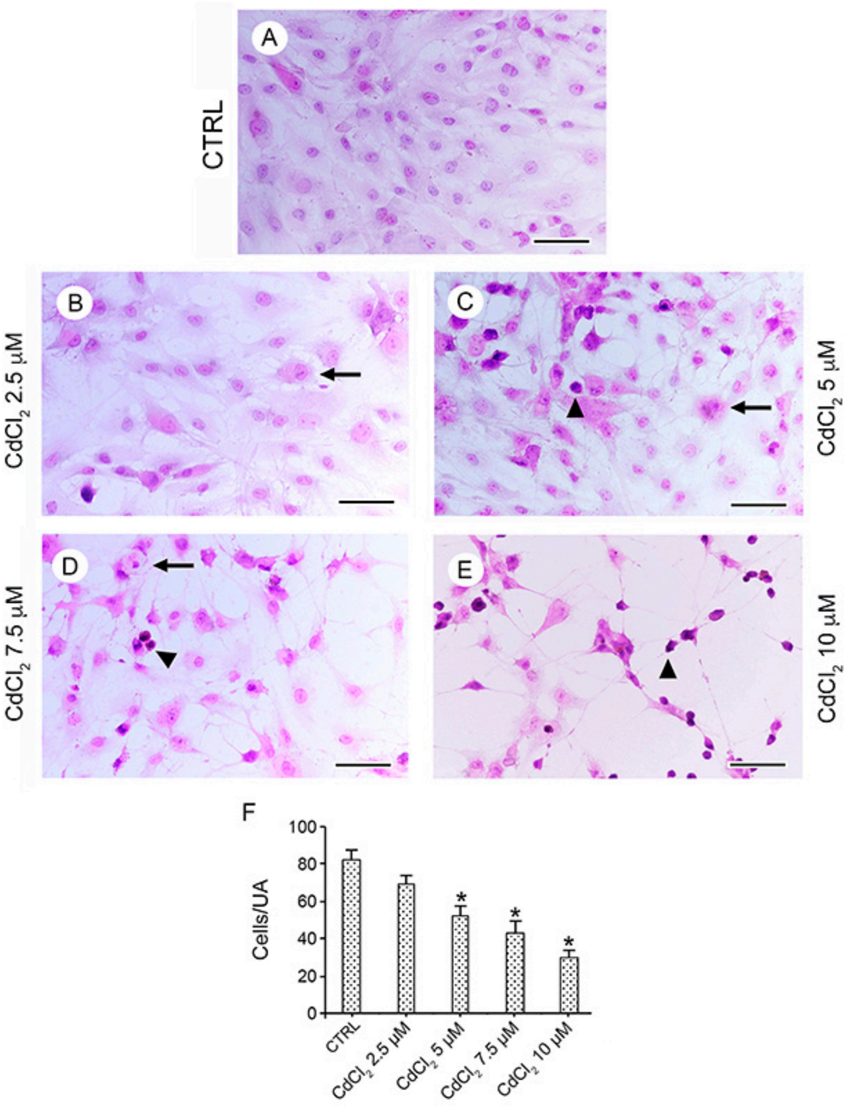


**Figure S3** – Histological organization of chondrocytes exposed to different doses of $CdCl_2$ and evaluated with hematoxylin-eosin stain (Scale bar: 100 μm). **A**: Control groups. Chondrocytes are regularly distributed and show uniform size, euchromatic nuclei and long processes. **B**: Chondrocytes challenged with 2.5 μM $CdCl_2$. Cells show irregular size and, occasionally, foamy cytoplasm (arrow). **C**: Chondrocytes challenged with 5 μM $CdCl_2$. Normal or necrotic (arrow) cells or cells with reduced size and heterochromatic nuclei (arrowhead) are present. **D**: Chondrocytes challenged with 7.5 μM $CdCl_2$. Cells are reduced in number, and normal, necrotic (arrow) or small with heterochromatic nuclei (arrowhead) cells are evident. **E**: Chondrocytes challenged with 10 μM $CdCl_2$. A lower number of cells is present. Few are normal, the other are small, with heterochromatic nuclei and thin processes (arrowhead). **F**: Histogram of chondrocytes/UA in the different groups (mean ± SD). * $p < 0.05$ versus CTRL.
